# We Tabulated and Organized American Board of Neurological Surgeons Primary Exam Keywords (2015-2023) so You Don’t Have to

**DOI:** 10.7759/cureus.39402

**Published:** 2023-05-23

**Authors:** Julian J Lin, Jeffrey Klopfenstein, Andres Maldonado, Todd McCall, Andrew Tsung, Dzung H Dinh

**Affiliations:** 1 Neurosurgery, University of Illinois College of Medicine at Peoria, Peoria, USA; 2 Neurological Surgery, University of Illinois College of Medicine at Peoria, Peoria, USA; 3 Neurosurgery, OSF Saint Francis Medical Center, Peoria, USA; 4 Neurosurgery, University of Illinois College of Medicine, Peoria, USA

**Keywords:** neurosurgery, neurosurgery residency, board exam, primary exam, american board of neurological surgeons

## Abstract

Background

Passing the American Board of Neurological Surgeons (ABNS) Primary Exam is required for residents in training. Both the program directors and residents are given keywords of the exam afterward in the hope to help program directors determine their relative strengths and weakness. We have organized and tabulated these keywords for neurosurgery residents’ benefit.

Methodology

We collected and analyzed ABNS Primary Exam keywords (2015-2023) in each of the exam’s main categories for trends and recurrences. We examined the overall passing rates among first-time credit test takers. The frequency of each subcategory was calculated as a percentage within its corresponding category. Recurrent keywords were grouped together with their corresponding years and categorized as once, twice, or thrice and greater occurrences; the last category was considered to be high-yield keywords.

Results

The number of questions in Neurosciences and Neurology has decreased over the years while Neurosurgery and Critical Care questions have increased. Similarly, there are fewer keyword repeats in Neurosciences and Neurology. The most repeated keywords are in Neuroimaging. The most common keywords are presented and listed along with the years of occurrences. Overall, the passing rate among first-time credit test takers is over 90%.

Conclusions

Neurosurgery residents can consider the common keywords as a guide in preparation for the ABNS Primary Exam.

## Introduction

The American Board of Neurological Surgery (ABNS) Primary Exam is administered during residency to test residents’ knowledge, and passing it along with the successful competition of residency are requirements toward board eligibility. The test consists of eight main categories for a total of 375 questions while some questions are not graded [1]. Every year, ABNS forwards test results along with graded test question keywords to program directors and residents. Program directors receive the complete list of keywords while residents receive keywords on missed questions. The goal is to help program directors determine the strengths and weaknesses of their program. There is very little written or published about these keywords. Similarly, the American Board of Surgery In-Training Exam (ABSITE) also distributes keywords to residents and program directors for their annual exam; however, the keywords in the ABSITE are much more general and less specific than those found in the ABNS [2]. We have tabulated and organized these keywords according to their occurrences to indicate high-yield contents in the hope to help residents study for the exam.

## Materials and methods

We collected and analyzed the ABNS item content keywords report for the Primary Exams from 2015 to 2023. In addition to keywords, the reports also contained Norm Tables, and passing rate among credit test takers can be calculated. Each set of keywords also contained percentages of correct among credit candidates and self-assessment test takers. Program directors receive the complete list of keywords while residents receive keywords on missed questions. We analyzed both sets of keywords.

We first calculated the total number of questions graded in each exam from 2015 to 2023 and looked for trends over the years. The keywords, already divided into eight categories, were then entered into a spreadsheet along with the corresponding year. In Neurosciences, we did not include the sub-subcategory listed by ABNS to simplify the list and to be consistent with other categories. Keywords are further subcategorized according to the ABNS content categories outline published in 2016 [1]. Repeated keywords were grouped together and highlighted when occurrences were three or greater. We took liberties to concise and group keywords leaving pertinent and specific information pertinent to the keywords intact. In Neuroimaging, we also made assignments that made the most sense as not all keywords included the type of imaging studies, which is the basis of ABNS categorization. For example, the authors assigned “Hydrocephalus, intraventricular hemorrhage, macrocephaly, Aqueductal stenosis” under Ultrasonography instead of CT or MRI.

We also calculated the frequency of its subcategories to determine the most tested areas in each category. Percentages of keyword occurrences were then calculated and divided into single-year occurrences, twice and thrice or more. Occurrences of three times or more were considered high yield and grouped together.

## Results

The number of questions in the eight categories tested each year is listed on the ABNS website: Neuroanatomy: 49, Neurosciences: 30, Neuropathology: 43, Neuroimaging: 57, Neurology: 40, Neurosurgery: 84, Critical Care: 65, and Core Competencies: 7 [1]. The total number of questions each year is 375. During 2015-2023, 4-17 questions out of 375 are not graded each year depending on the year. The number of test questions decreased in Neurosciences and Neurology from 2016 to 2017 while Neurosurgery and Critical Care increased. Table 1 shows the frequency of each subcategory in the Primary Exam tested during 2015-2023 in percentages.

**Table 1:** Frequency of each subcategory in the Primary Exam tested during 2015-2023 in percentages. ABNS: American Board of Neurological Surgeons; BBB: blood-brain barrier; CSF: cerebrospinal fluid; EMG: electromyography; NCV: nerve conduction study; EEG: electroencephalography

ABNS categories and subcategories	Percentage
Neuroanatomy	
Embryology	8.8
Cerebrum	12
Cerebellum	3.4
Basal ganglia	4.9
Thalamus	4.4
Hypothalamus and pituitary	4.4
Brain stem	5.1
Cranial nerves	9.5
Spinal cord	2.9
Nerve root	0.2
Plexi	3.9
Peripheral nerve	9.8
Muscle and peripheral receptor	2.4
Vascular (including BBB)	9.8
Cytoarchitecture and subcellular anatomy	3.2
CSF pathway	3.2
Skeletal (including skull)	4.6
Meninges	0.5
Autonomic	3.4
Other	3.6
Neurosciences	
Neurons and axons	31
Muscles	11.5
Pharmacology	8.8
Functional systems	16.2
Glia	0
Cerebral vascular system	9.6
Responses to injury	1.5
Nervous system development and plasticity	1.2
Cerebrospinal fluid	3.1
Spine	3.1
Cell and viral biology	14.2
Neuropathology	
Developmental and genetic	18.6
Vascular	18.4
Neoplastic	33.9
Traumatic	4
Infectious	6.2
Metabolic	2.8
Demyelinating	2.5
Degenerative	8.5
Neuromuscular	2.8
Toxic	0.6
Other	1.7
Neuroimaging	
Radiography	11.9
Angiography	20
Myelography	0.2
Computed tomography	15.1
Magnetic resonance imaging	45.3
Radioisotope imaging	2
Ultrasonography	0.8
Positron emission tomography	4.2
Other	0.4
Neurology	
Epilepsy	10.5
Demyelinating	7
Degenerative	5.5
Behavioral	1.5
Developmental	11.4
Infection	4.1
Metabolic	4.4
Endocrine	2.9
Electrical Studies, EMG, NCV, and EEG	7.6
Muscle and Nerve	9.9
Pain	4.4
Pharmacology	4.1
Toxic	2
Neuro-ophthalmology	3.5
Neurologic signs and examinations	3.5
Vascular	7.9
Movement disorders	5
Genetic	1
Autoimmune	2.9
Other	1.5
Neurosurgery	
Cranial - Infection	2.5
Cranial - Congenital	7
Cranial - Trauma	4.5
Cranial - Tumor	11.3
Cranial - Vascular	9.1
Cranial - Other	2
Extracranial vascular	3.2
Spinal - Congenital	1.8
Spinal - Degenerative	6.4
Spinal - Infection	1.2
Spinal - Trauma	10
Spinal - Tumor	2.6
Spinal - Vascular	1.8
Spinal – Biomechanics	1.5
Spinal - Deformity	1.9
Spine - Other	0.1
Complications - Cranial and spinal	3.4
Cranial nerves	1.2
Peripheral nerve	5.3
Pain	6.1
Endocrine	2.6
Surgical technique	0.7
Autonomic	0.7
Pharmacology	0.7
Movement disorders	3.2
Radiosurgery	2.6
Endovascular	3.2
Surgical epilepsy	3.2
Other	0.5
Critical care	
Fluid and electrolytes	6.3
Shock	6.5
Cardiovascular	7.5
Multiple trauma	12.5
Hematology and coagulopathies	11.1
Nutrition	1.5
Pulmonary	13.2
Endocrine	6.5
Gastrointestinal	0.8
Genitourinary	1.7
Pharmacologic	9.4
Infection and immunologic	5.4
Toxicology	3.3
Wound healing	2.7
Anesthesia	2.3
Cerebral metabolism	7.1
Other	2.1

Neuroanatomy

Neuroanatomy is mostly categorized according to anatomical structures. Embryology, cerebrum, cranial nerves, peripheral nerve, and vascular/including blood-brain barrier (BBB) are the most common subcategories tested. Approximately 12.9% of keywords are tested only once in this category, 23.4% twice, and 63.7% thrice or more during this time period. Occurrences of three times or more are considered high yield. Table 2 shows the details for high-yield keywords and occurrences. The same topics/keywords may be tested multiple times during the same year and their occurrences are grouped together. The most highly tested topics in Neuroanatomy include the following keywords: septal vein, foramen of Monro, fornix, thalamostriate vein, lamina terminalis, third ventricle, infundibular recess, choroid plexus, and hydrocephalus. The residents were likely asked to identify these anatomical structures during an endoscopic procedure in a patient with hydrocephalus.

**Table 2:** High-yield keywords and occurrences in Neuroanatomy.

Neuroanatomy keywords	Occurrence years
Embryology	
Occult dysraphism, dysjunction	2016, 2020, 2022
Dermal sinus tract, anterior neuropore, embryology, neurulation, tethered cord	2016, 2018, 2019, 2020, 2022
Embryology, myelomeningocele	2016, 2017, 2020, 2022
Alar plate, basal plate, sulcus limitan	2015, 2016, 2021, 2022
Metencephalon, embryology	2019, 2020, 2022
Olfactory/limbic system--development, cortical surface--olfaction	2020, 2022, 2022
Lamina terminalis, anterior end of the neural tube, primary neurulation, folding	2016, 2020, 2022, 2023
Cerebrum	
Cerebral cortex, cortical layers, corticocortical association connections	2020, 2022, 2023
Supplementary motor area, cortical mapping, motor control, blood supply	2017, 2018, 2019
Uncinate fasciculus, white matter fiber tracts	2017, 2018, 2019, 2020, 2021, 2021, 2022
Amygdala, efferent pathway/amygdaloid complex, anterior commissure	2015, 2016, 2017, 2020, 2020, 2022, 2022
Calcarine sulcus, cuneus, color vision, blindness, neuropsychology, occipital lobe	2016, 2019, 2020, 2022
Marginal limb or cingulate sulcus	2015, 2019, 2020, 2022
Temporal lobe, parahippocampal, fusiform, hippocampal formation, subiculum	2015, 2020, 2021, 2023
Cerebellum	
Purkinje cells (dendritic arborization), excitatory cells, granule cells, mossy, climbing fiber origin	2015, 2017, 2018, 2019, 2023
Cerebellar lesion, tremor, tract, dentatorubrothalamic, dentate nucleus, dentatothalamic tract	2015, 2016, 2016, 2017, 2023
Basal ganglia	
Medial lemniscus (somatotopic, lesion), subthalamic nucleus (afferents), blood supply	2015, 2015, 2016, 2017, 2017, 2017, 2018, 2019, 2021
Substantia nigra, thalamus, basal ganglia, brain vesicle, subthalamic nucleus, Parkinson’s, dopamine	2015, 2015, 2016, 2016, 2016, 2017, 2017, 2021
Thalamus	
Sensory nucleus of thalamus, ventroposterior nucleus, post-central gyrus, VPL, homunculus	2015, 2016, 2019, 2020, 2022
Internal capsule (infarct), thalamus, ventral medial nucleus	2015, 2016, 2017, 2019
Thalamic nuclei (projection), Hassler, tremor	2016, 2016, 2017, 2018, 2019
Brainstem	
Brainstem anatomy, spinal tracts, somatic sensation, functional columns	2020, 2021, 2022
Floor of the fourth ventricle, stria medullaris, choroid plexus, facial colliculus	2017, 2017, 2019, 2020, 2022
Cranial nerves	
Vagal nerve, hoarseness, dysphagia, superior laryngeal nerve, glossopharyngeal ganglion, carotid artery pressure	2015, 2016, 2016, 2018
Trigeminal nucleus, trigeminal nerve, trigeminothalamic tract, trigeminal relay nuclei, lacrimal reflex	2015, 2016, 2018, 2018, 2023
Solitary nucleus, geniculate ganglion (taste), sensation	2015, 2020, 2022
Olfactory tract, axons mitral and tufted cells, olfaction	2015, 2016, 2019
Facial nerve, acoustic surgery, GSPN, autonomic, hearing, hyperacusis	2015, 2016, 2016, 2016, 2019, 2019, 2021
Trigeminal nerve, meninges, dura, innervation, middle cranial fossa, rhizotomy, skull, pain, foramen ovale	2018, 2020, 2022, 2023
Spinal cord	
Spinal cord, dorsal columns, pyramidal tract, functional anatomy, alar plate	2015, 2017, 2017, 2018
Spinothalamic tract, spinal cord, somatosensory, Lissauer's tract	2015, 2016, 2017, 2017
Plexi	
Brachial plexus, erb palsy, upper trunk, lower trunk	2015, 2015, 2015, 2016, 2016, 2016, 2016, 2017, 2017, 2018, 2021, 2023
Peripheral nerve	
Dorsal scapular nerve, rhomboid, winged scapula, long thoracic nerve	2015, 2016, 2016 2023
Ulnar nerve innervated muscles, first dorsal interosseous muscle, sensation, hand, forearm	2017, 2019, 2020, 2022, 2023
Axillary nerve	2015, 2021, 2023
Radial nerve, wrist drop, dorsal cutaneous nerve, forearm, supinator entrapment, PIN, interosseous muscle function	2015, 2015, 2015, 2016, 2016, 2016, 2017 2019, 2021, 2021, 2023
Ulnar neuropathy, cubital tunnel, pisohamate ligament	2015, 2018, 2021, 2023
Peroneal nerve, foot drop	2015, 2019, 2023
Muscle and peripheral receptor	
Type 1 muscle fibers, muscle spindle, muscle physiology	2015, 2016, 2016, 2016, 2017, 2019, 2023
Innervation, bladder afferent, detrusor muscle	2017, 2019, 2021
Vascular (including blood-brain barrier)	
BBB	2016, 2016, 2019
Angiogram, meningohypophyseal trunk, petroclival meningioma	2016, 2017, 2018, 2019, 2019
Internal auditory canal/artery, AICA, acoustic neuroma surgery	2015, 2017, 2021
Anterior choroidal artery, stroke, IC/ant limb, globus, caudate, lenticulostriate	2017, 2019, 2021, 2023
Inferior petrosal sinus, petrosal vein microvascular decompression	2018, 2019, 2020
Cytoarchitecture and subcellular anatomy	
Melanocytes, CNS location, melanin, neuron, locus ceruleus	2016, 2017, 2023, 2023
Cerebrospinal fluid pathway	
Septal vein, foramen of Monro, fornix, thalamostriate vein, lamina terminalis, third ventricle, infundibular recess, choroid plexus, hydrocephalus	2015, 2015, 2015, 2016, 2016, 2017, 2018, 2020, 2020, 2020, 2021, 2022, 2022, 2022, 2022, 2023, 2023
Skeletal (including skull)	
Dens, odontoid, development, embryology, os odontoideum, C1, embryology	2016, 2016, 2016, 2018, 2017, 2019, 2020, 2022, 2023
Autonomic	
Autonomic nervous system anatomy, vidian nerve	2016, 2018, 2021
Adrenal medulla	2018, 2020, 2022
Sympathetic preganglionic neurons	2015, 2015, 2016, 2023, 2023

Neurosciences

Neurosciences, sometimes labeled neurobiology, is divided among microstructures, functional systems, and neurophysiology. We did not include the sub-subcategory listed by the ABNS to simplify the list and to be consistent with other categories. Approximately 18.1% of keywords are tested only once in this category, 23.8% twice, and 58.1% thrice or more during this time period. Table 3 shows the details for high-yield keywords and occurrences. There are overlaps with other categories found in keywords such as cerebral ischemia, diffusion-weighted imaging, and MR imaging. The most highly tested topics in Neurosciences include the following keywords: acetylcholine, cholinergic output, cholinergic nerve function, anticholinergic, muscarinic receptor, antagonists, and enzymatic degradation.

**Table 3:** High-yield keywords and occurrences in Neurosciences.

Neurosciences keywords	Occurrence years
Neurons and axons	
Motor neuron, spinal cord dorsal horn, nociceptive afferents, glutamate, molecular induction, interneuron, inhibition, Renshaw cells	2015, 2015, 2015, 2016, 2017, 2018, 2018, 2019, 2019, 2019
Axons and neurons ‐ transmitters, postsynaptic response to transmitter	2015, 2017, 2018, 2020, 2022
Dopamine, limbic loop, nucleus accumbens, ventral tegmental area, Basal ganglia, synapses, synaptic vesicles, amino acids	2015, 2016, 2017, 2018, 2020, 2020, 2022, 2022 2023
GABAergic neurons, inhibitory neurotransmitter, GABA modulator	2016, 2017, 2018, 2019
Synapse, calcium, ion channels, transmitters, cell biology, channels	2015, 2017, 2018, 2021, 2023
Neuronal synapse, neuronal structure, electrical properties, synaptic delay	2017, 2020, 2021
Acetylcholine, cholinergic output, cholinergic nerve function, anticholinergic, muscarinic receptor, antagonists, enzymatic degradation	2015, 2016, 2016, 2018, 2018, 2018, 2020, 2020, 2021, 2022, 2022, 2023
Glutamate excitotoxicity, NMDA glutamate receptor, magnesium	2017, 2020, 2022, 2023
Membrane, K+ channels, nerve action potential--K+ permeability, K+ channels--drug action	2017, 2020, 2022
Axonal action potential, membrane potential, peak action potential determinant	2015, 2016, 2020, 2022, 2023
Motor evoked potentials, intraoperative monitoring, caudal D wave, SSEP dorsal columns	2020, 2022, 2023
Muscles	
Stretch reflex synapses, afferent reflex fibers, Ia axons, muscle stretch reflex, neurotransmitters, myotactic reflex	2015, 2017, 2018, 2019
Hoffman reflex, sensor fibers, H reflex, electromyography	2015, 2017, 2020, 2022
Mechanoreceptors, Meissner corpuscle, Merkel disk, Merkel cell afferents	2015, 2016, 2020, 2022, 2023
Muscle spindle, Golgi tendon organ, Golgi complex--function	2015, 2019, 2019, 2020, 2022
Tau, microtubules	2016, 2018, 2023
Pharmacology	
Curare, muscle paralysis	2015, 2017, 2018, 2019, 2020, 2022
Dantrolene, malignant hyperthermia, anesthesia, muscle anatomy	2016, 2018, 2019, 2023
Neuromuscular junction, succinylcholine	2015, 2016, 2018
Functional systems	
Visual system, ganglion cells, optic nerve, utricular macula, night blindness, vitamin A deficiency	2018, 2019, 2020, 2022, 2023
Hearing loss, BAER waveform component--location, auditory translation--basilar membrane, absence of wave V	2015, 2019, 2020, 2022, 2023
Sensory nerve, parasympathetic nervous system, facial nerve	2015, 2015, 2016, 2016
Cerebral vascular system	
Cerebral blood flow, traumatic brain injury, cerebral blood flow measurement, autoregulation	2015, 2018, 2020, 2022, 2023
Seizures--cerebral blood flow, oxygen	2015, 2018, 2019
Blood-brain barrier--molecule passage, anatomic basis, permeability	2015, 2015, 2016, 2017, 2018, 2021, 2023
Cerebral ischemia, diffusion-weighted imaging, MR imaging	2019, 2019, 2023
Cerebrospinal fluid	
CSF absorption, CSF production	2015, 2015, 2018, 2019, 2020, 2022
Cell and viral biology	
Cowden disease, Lhermitte-Duclos disease, PTEN gene	2015, 2017, 2018
Reflex sympathetic dystrophy, complex regional pain syndrome	2019, 2020, 2022
Progressive supranuclear palsy, Tau immunoreactive protein	2017, 2020, 2022
Huntington disease‐‐neurochemistry, acetylcholine transferase	2020, 2022, 2023

Neuropathology

Residents are frequently asked to identify structures or name disorders based on histopathological slides/micrographs/photos in Neuropathology. Approximately 11.6% of keywords are tested only once in this category, 11.2% twice, and 77.2% thrice or more during this time period. Table 4 shows the details for high-yield keywords and occurrences. The most highly tested topics in Neuropathology include the following keywords: von Hippel-Lindau disease, hemangioblastoma, and stromal cells.

**Table 4:** High-yield keywords and occurrences in Neuropathology.

Neuropathology keywords	Occurrence years
Developmental and Genetic	
Sinus pericranii, vascular malformations, infancy	2016, 2020, 2022
Epidermoid tumor pathology, common location	2018, 2020, 2021, 2022
Neurenteric cyst, embryology, histology, intradural lesion, spinal mass	2015, 2016, 2023
Apert syndrome, FGFR2 gene mutation, syndactyly, exophthalmos	2018, 2021, 2023, 2023
Findings/leukodystrophies, metachromatic leukodystrophy	2020, 2022, 2023
Epilepsy, mesial temporal lobe epilepsy, hippocampal sclerosis	2017, 2019, 2020, 2020, 2022, 2022
Cortical dysplasia, epilepsy, balloon cells	2016, 2021, 2023
Hypothalamic hamartoma	2017, 2020, 2022
Proteins associated with NF2, vestibular schwannoma, phakomatoses, neurocutaneous syndromes	2015, 2018, 2019, 2023, 2023
Tuberous sclerosis, subependymal giant cell astrocytoma, ventricular tumor	2016, 2020, 2022, 2023
Von Hippel-Lindau disease, hemangioblastoma, stromal cell	2015, 2015, 2016, 2016, 2018, 2019, 2019, 2020, 2020, 2020, 2022, 2022, 2023, 2023
Sturge-Weber/vascular malformation	2018, 2018, 2019, 2020, 2021, 2022, 2023
Vascular	
Carotid plaque, symptomatic, atherosclerosis, pathophysiology, Ischemic stroke, carotid stenosis, carotid stenting	2016, 2017, 2018, 2019, 2021, 2023
Hypertensive intracerebral hemorrhage, postoperative complications	2015, 2016, 2016, 2017, 2018, 2019, 2021, 2023
Cavernous malformation, developmental venous anomaly, angiographic appearance, endothelial cells, genetic predisposition, familial occurrence	2018, 2019, 2019, 2020, 2021, 2021, 2022, 2023, 2023
Brain hypoxia, pseudolaminar necrosis, hypoxic ischemic encephalopathy	2015, 2015, 2017, 2018, 2021
Arteriovenous malformation, cerebellar hemorrhage	2016, 2019, 2021, 2023
Cerebral aneurysm, pathophysiology, superior hypophyseal artery aneurysm, cerebrovascular anatomy, aneurysm formation, vessel wall	2017, 2020, 2020, 2022
Moyamoya disease	2019, 2020, 2022
Cerebral amyloid angiopathy, intracerebral hemorrhage	2016, 2017, 2019, 2019
Neoplastic	
Lymphoma, central nervous system, pathology, large B-cell lymphoma	2020, 2022, 2023
Primary glioblastoma, genomic analysis, p53, Li‐Fraumeni, Genetic profile and favorable prognosis in a glioma	2016, 2018, 2020, 2020, 2021, 2022, 2022
IDH mutation with 1p19q co-deletion, glioma	2020, 2021, 2021
Oligodendroglioma, 1p/19q deletion, PTCH1, p53 deletion	2015, 2017, 2017, 2018, 2020, 2021, 2022
Hemangiopericytoma, immunohistochemistry	2015, 2016, 2017, 2018, 2020, 2022, 2022
Meningiomas--chromosome deletions, histopathology, immunohistochemistry, MR spinal mass, H&E stain	2015, 2016, 2016, 2018, 2020, 2020, 2021, 2022, 2022, 2023, 2023
Vestibular schwannoma, Antoni A region, Verocay bodies, nerve sheath tumor	2019, 2019, 2021, 2023, 2023
Ganglioglioma--photomicrograph	2015, 2020, 2022
Ependymoma, pseudorosettes, true rosettes, chicken-wire vasculature, location	2015, 2019, 2021, 2023, 2023
Medulloblastoma, posterior fossa tumor, cerebellum, pediatric, brain tumor, molecular pathology, Gorlin syndrome	2017, 2018, 2021, 2023, 2023
Pilocytic astrocytoma, Rosenthal fibers	2015, 2016, 2016, 2019, 2020, 2020, 2021, 2022, 2022, 2023, 2023
Cisplatin toxicity	2015, 2016, 2017, 2018
Diffuse pontine glioma, K27M mutation, Histone mutation, thalamic tumor, pediatric brain tumor, prognosis, midline tumor	2018, 2020, 2020, 2021, 2022, 2022, 2023
Craniopharyngioma, supra-sellar, tumor, pediatric, pathology, adamantinomatous	2018, 2020, 2022, 2023
Vertebral hemangioma, intraosseous vascular stroma, “polka dot” or “salt and pepper”	2017, 2020, 2022
Myxopapillary ependymoma, perivascular pseudorosettes, hyaline mucinous material, spinal cord tumor, ependymoma	2016, 2017, 2017, 2017, 2020, 2022
Adenocarcinoma, metastasis to spine, histopathology	2015, 2015, 2016
H&E stains, spinal cyst, CT myelogram, synovial cyst, myxoid degeneration	2017, 2020, 2022
Traumatic	
Tau protein, chronic traumatic encephalopathy	2016, 2018, 2018, 2019
Head injury, diffuse axonal injury	2015, 2016, 2017, 2018
Infectious	
Candida, brain abscess, pseudohyphae	2015, 2017, 2018
HIV, CNS infection, toxoplasmosis	2017, 2018, 2021
Common brain abscess organisms	2016, 2017, 2018, 2021
PML etiology	2017, 2018, 2021
Metabolic	
Wernicke encephalopathy, mammillary bodies, nutritional disorder	2017, 2018, 2018, 2019
Sarcoidosis, histopathology	2015, 2016, 2019
Demyelinating	
Multiple sclerosis plaque, T cells, macrophage staining, demyelination, tumefactive	2015, 2016, 2017, 2017, 2018, 2018, 2021
Degenerative	
Subacute combined degeneration pathology	2015, 2016, 2017, 2018
Amyotrophic lateral sclerosis	2015, 2016, 2016, 2017, 2019
Neurofibrillary tangles, Alzheimer’s disease, Tau protein, basal forebrain, cholinergic	2016, 2020, 2021, 2022
Parkinson’s disease, Lewy bodies, protein, Lewy neurites, alpha‐synuclein	2018, 2018, 2019, 2020, 2020 2022, 2022, 2023
Caudate/putamen atrophy, Huntington	2015, 2016, 2019
Neuromuscular	
Charcot-Marie-Tooth, demyelination	2015, 2016, 2018, 2019
Peripheral neuropathy, neurogenic atrophy	2016, 2017, 2018, 2019
Other	
MR image, H&E stain, fever, neoplasms, immunohistochemistry	2020, 2020, 2022, 2022

Neuroimaging

Neuroradiology is categorized based on imaging modalities and relies heavily on images. Approximately 7% of keywords are tested only once in this category, 10.8% twice, and 82.2% thrice or more during this time period. Table 5 shows the details for high-yield keywords and occurrences. The authors took the liberty to make assignments that made the most sense as not all keywords included the type of imaging studies, which is the basis of ABNS categorization. For example, the authors assigned “Hydrocephalus, intraventricular hemorrhage, macrocephaly, Aqueductal stenosis” under Ultrasonography instead of CT or MRI. The most highly tested topics in Neuroradiology include the following keywords under angiography: cavernous malformation, developmental venous anomaly, and venous angioma.

**Table 5:** High-yield keywords and occurrences in Neuroimaging.

Neuroimaging keywords	Occurrence years
Radiography	
Hydrocephalus, ventriculoperitoneal shunt, shunt malfunction, shunt series	2017, 2017, 2020, 2021, 2022
Leptomeningeal cyst, growing skull fracture	2015, 2017, 2018, 2020, 2021
Calcified cephalohematoma, expanding skull fracture	2016, 2016, 2017, 2021, 2022
Plain X-ray, orbital defect	2015, 2016, 2017, 2019, 2023
Osteoid osteoma	2017, 2018, 2020
Diagnose from intracranial calcification, normal intracranial calcification	2015, 2016, 2017, 2020, 2020, 2022, 2022
Cervical spine, dens/atlas distance, flexion teardrop fracture, unstable fracture	2019, 2020, 2022, 2023
Spinopelvic, pelvic incidence, sagittal imbalance	2016, 2017, 2018, 2020, 2022
Diffuse idiopathic skeletal hyperostosis (DISH), vertebral hyperostosis, ankylosing spondylitis, Marie-Strumpell--X-ray diagnosis	2015, 2016, 2016, 2018
Isthmic spondylolisthesis, pediatric back pain, spondylolysis, pars defect	2016, 2017, 2023
Angiography	
Cerebral angiography, tumor	2015, 2016, 2018, 2019, 2021
Moyamoya syndrome, stroke, transient ischemic attack	2018, 2020, 2022
DAVF, occipital artery, fistula, venous anatomy	2020, 2021, 2022, 2022, 2020, 2023
Carotid-cavernous fistula, anatomy, superior ophthalmic vein, sequelae	2015, 2016, 2017, 2018, 2019, 2020, 2021, 2021, 2022, 2023, 2023, 2023
Vein of Galen malformation, arteriovenous fistula, aneurysm	2018, 2019, 2020, 2020, 2022, 2022, 2023
Arteriovenous malformation, cortical anatomy, PICA, AVM, aneurysm, SAH	2015, 2017, 2020, 2020, 2021, 2022, 2022
Middle cerebral artery aneurysm, Unruptured aneurysm	2017, 2020, 2021, 2022, 2023
Carotid aneurysm, basilar bifurcation aneurysm; subarachnoid hemorrhage	2020, 2021, 2022, 2023
Anterior cerebral artery, pericallosal aneurysm, pericallosal artery	2017, 2018, 2018, 2019, 2019, 2020, 2021, 2022
Cerebrovascular anatomy, ascending pharyngeal artery	2019, 2020, 2022
Anterior choroidal artery, posterior cerebral artery, p-comm, aphasia, carotid artery	2017, 2018, 2019, 2019, 2019, 2021
Circle of Willis, variation	2015, 2016, 2016, 2017, 2020, 2021, 2022, 2023
Vein of Labbe, venous anatomy, Internal cerebral vein	2019, 2023, 2023
Congenital anomalies, proatlantal intersegmental artery, persistent embryonic arteries	2017, 2017, 2017, 2018, 2023
Anterior spinal artery, spinal arteriovenous malformation, spinal angiography	2017, 2019, 2019, 2021, 2023
Hemangioblastoma, spinal angiogram, tumor blush, Intramedullary cervical cord tumor	2016, 2016, 2018, 2019, 2023
Computed tomography	
Temporal bone anatomy, bone windows, skull base	2016, 2017, 2018, 2021, 2023
Ischemic stroke, Weber syndrome, CT perfusion scan, cerebral edema, mid-cerebral artery, tissue plasminogen activator, mechanical thrombectomy	2016, 2016, 2017, 2017, 2019, 2021, 2022, 2023, 2023
CTA, aortic arch anatomy, posterior circulation	2015, 2015, 2016, 2016, 2017, 2019, 2021, 2023
Amyloidosis, amyloid angiopathy, intracerebral hemorrhage, volume measurement, spot sign, prognostic factors	2016, 2021, 2021, 2023
Metopic suture synostosis, craniosynostosis	2018, 2020, 2022
CT scan, epidermoid cyst	2016, 2017, 2018, 2019, 2023
Pagets disease, skull	2015, 2020, 2022
Eosinophilic granuloma, skull X-ray films, CT scan, Langerhans cell histiocytosis	2015, 2017, 2020, 2020, 2021, 2022, 2022
Fibrous dysplasia, bone lesion	2015, 2016, 2018, 2019, 2020, 2021, 2022, 2023
Ventriculitis on CT--child	2017, 2018, 2020, 2022
Osteoid osteoma, spine lesion	2015, 2016, 2019, 2022, 2023
Rotary subluxation, cervical spine trauma	2015, 2020, 2022
Ossification of the posterior longitudinal ligament, cervical stenosis, cervical myelopathy	2016, 2020, 2022
Magnetic resonance imaging	
Proton MR Spectroscopy interpretation, glioma,, glioblastoma	2016, 2019, 2021, 2021
Meningioma, olfactory groove	2015, 2019, 2021
MR scan, trigeminal nerve	2017, 2018, 2020, 2022, 2023
Hemifacial spasm, vertebral artery, vessel visualization	2015, 2015, 2020, 2022
Medulloblastoma--identify on MR scan, MR spectroscopy	2015, 2018, 2019, 2021
Pilocytic astrocytoma, optic glioma, cerebellar astrocytoma	2015, 2015, 2016, 2016, 2017, 2021, 2023
Pituitary apoplexy, pituitary tumor, pituitary hyperplasia, prolactin	2015, 2017, 2017, 2019, 2019, 2019, 2020, 2020, 2021, 2022, 2022, 2023, 2023
Rathke’s cyst	2015, 2016, 2023
Schwannoma, cranial nerve, skull base tumors, Trigeminal schwannoma	2016, 2016, 2018, 2021
Neurocytoma, intraventricular tumors	2017, 2018, 2021, 2022
Pineal cyst, endoscopy, pineal region surgery, pineal region tumor	2016, 2017, 2018, 2019, 2019, 2023
Germ cell tumor, germinoma	2015, 2016, 2018, 2019
Craniopharyngioma, histology	2015, 2015, 2016, 2018, 2021
Epidermoid tumor, dermoid, fourth ventricle	2016, 2017, 2019, 2021
Multiple sclerosis, demyelination, tumefactive, ring-enhancing lesion	2015, 2016, 2018, 2019, 2019
PRES, posterior reversible encephalopathy syndrome	2016, 2017, 2019
Demyelinating disease vs. white matter lesions, adrenoleukodystrophy	2020, 2022, 2023
Tuberous sclerosis, subependymal giant cell astrocytoma, neurocutaneous syndrome	2015, 2016, 2017, 2023
Phakomatosis, autosomal dominant, hemangioblastoma, von Hippel-Lindau disease, Retinal angioma, ataxia	2016, 2019, 2021, 2023, 2023
Early cerebral infarction, diffusion-weighted imaging, acute brain ischemia	2015, 2020, 2021, 2022
Cavernous malformation, developmental venous anomaly, venous angioma	2015, 2015, 2015, 2015, 2016, 2016, 2016, 2017, 2017, 2018, 2019, 2019, 2020, 2021, 2022, 2023, 2023
Internal cerebral vein, vascular anatomy	2015, 2018, 2021, 2023, 2023
Chiari malformation, syringomyelia, CSF circulation	2015, 2018, 2019, 2020, 2022
CSF hypotension, pachymeningeal enhancement, Spontaneous intracranial hypotension	2016, 2018, 2019, 2020, 2021, 2022
Empty sella syndrome	2015, 2016, 2017
Neurocysticercosis, ring enhancing lesion	2018, 2020, 2021, 2022
Subdural empyema; infection, mucocele, frontal abscess	2015, 2021, 2023
Progressive multifocal leukoencephalopathy, JC virus, immunosuppression	2016, 2017, 2018, 2019
Brain abscess, diffusion-weighted MRI	2016, 2016, 2017, 2018, 2018
Tethered spinal cord, filum terminale lipoma	2015, 2015, 2018, 2023
Synovial cyst	2015, 2018, 2019, 2021, 2023
Hematogenous osteomyelitis, discitis, epidural abscess	2018, 2019, 2021, 2021, 2023
Osteochondroma, spinal tumor	2018, 2020, 2022
MR image: spinal chordoma	2018, 2020, 2022
Metastasis to spine, spinal instability	2015, 2017, 2018, 2020, 2022
Lateral disc herniation	2019, 2020, 2022
Radioisotope imaging	
Epilepsy, SPECT imaging, mesial temporal sclerosis, Tumor	2015, 2015, 2015, 2016, 2017, 2017, 2021
Ultrasonography	
Hydrocephalus, intraventricular hemorrhage, macrocephaly, aqueductal stenosis	2015, 2016, 2021
Positron emission tomography	
PET scan, recurrent tumor, radionecrosis, Stereotactic radiosurgery, tumor recurrence, MR spectroscopy	2016, 2017, 2018, 2019, 2019, 2021
PET imaging, imaging of cellular biological activity	2016, 2019, 2021
Glioma, [11C] methionine PET, PET, high-grade glioma	2015, 2018, 2020, 2021, 2022
Interictal epilepsy, FDG-PET scanning, metabolism, [18F] fluorodeoxyglucose PET	2015, 2017, 2019, 2021, 2023

Neurology

Overall, Neurology does not have a consistent trend of repeating keywords or topics as it is such a wide field. Approximately 19% of keywords are tested only once in this category, 22% twice, and 59% thrice or more during this time period. Table 6 shows the details for high-yield keywords and occurrences. The most highly tested topics in Neurology include the following keywords: EMG, foot drop, L5 radiculopathy, common peroneal neuropathy, extensor digitorum brevis, and femoral neuropathy.

**Table 6:** High-yield keywords and occurrences in Neurology.

Neurology keywords	Occurrence years
Epilepsy	
Anticonvulsants, suicidal ideation, pharmacology, epilepsy, hepatic failure	2015, 2016, 2020, 2022, 2022
Lennox-Gastaut, drop attacks, EEG findings, Callosotomy, atonic seizures	2015, 2016, 2016, 2020, 2022, 2023
Epilepsy surgery, mesial temporal sclerosis, medically refractory epilepsy	2018, 2020, 2022
Absence seizures, anticonvulsants, ethosuximide	2017, 2018, 2020, 2020
Hypothalamic hamartoma, gelastic seizures, intractable epilepsy	2017, 2018, 2019
Status epilepticus	2015, 2015, 2019, 2020, 2022, 2022
Rasmussen encephalitis	2015, 2016, 2023
Demyelinating	
Demyelinating disease	2020, 2022, 2023, 2023
Neuromyelitis optica, antibodies, optic neuritis, demyelination	2015, 2015, 2017, 2019
Multiple sclerosis, evoked potentials, symptoms and signs	2015, 2016, 2016, 2017, 2023
MLF, internuclear ophthalmoplegia, multiple sclerosis, extraocular movement	2016, 2017, 2020, 2022
Degenerative	
Alzheimer's disease—Down’s syndrome	2016, 2019, 2023
Neuroleptic sensitive dementia, Lewy bodies, neuroleptic therapy	2016, 2018, 2019
Wernicke's disease--triad of symptoms, alcoholism	2015, 2016, 2017, 2020, 2022
Charcot joint--etiology	2015, 2016, 2018, 2021
Behavioral	
Obsessive-compulsive disorder, behavioral disorder, functional neurosurgery	2018, 2020, 2021, 2022
Developmental	
Development, infant, neurological examination, pediatrics	2020, 2021, 2022
Myelomeningocele, folate, dietary deficiency, Diastematomyelia, dysraphism	2015, 2016, 2017
Sturge-Weber syndrome, genetics	2017, 2020, 2022
Neurofibromatosis, phakomatoses, Cafe au lait spots, genetics	2015, 2015, 2016, 2017, 2020, 2021, 2022
Crouzon's disease, features of Apert syndrome	2020, 2022, 2023
Tuberous sclerosis, neurocutaneous syndromes, seizures, retinal hamartoma	2015, 2016, 2019, 2023
Infection	
Herpes simplex encephalitis, Ramsay-Hunt syndrome, cranial nerves	2018, 2019, 2020, 2022, 2023
AIDS, HIV, CD4, toxoplasmosis, CNS complications in AIDS	2015, 2017, 2023
Metabolic	
Vitamin deficiency dementia, nutrition	2016, 2020, 2022
Combined systems disease, vitamin B12, proton pump inhibitors	2015, 2016, 2020, 2022, 2023
Endocrine	
Pituitary tumor, endocrine, prolactinoma	2015, 2017, 2018
Nelson's syndrome--findings	2018, 2020, 2022
Electrical studies, including EMG, NCV, EEG, etc.	
EMG, peripheral nerve injury, Erb's point, median SSEP	2015, 2016, 2016, 2017, 2018, 2019, 2023
EMG, foot drop, L5 radiculopathy, common peroneal neuropathy, extensor digitorum brevis, Femoral neuropathy	2015, 2016, 2016, 2018, 2019, 2020, 2021, 2021, 2022, 2023
Amyotrophic lateral sclerosis, nerve conduction block, riluzole	2017, 2017, 2018, 2019, 2021
Muscle and nerve	
Femoral neuropathy, peripheral diabetic neuropathy, treatment	2015, 2016, 2017, 2019, 2019, 2023
Duchenne muscular dystrophy, muscle disease	2015, 2017, 2019
Diabetic mononeuropathy, Third nerve palsy	2018, 2023, 2023
Polyneuropathy, chronic renal disease	2016, 2017, 2020, 2022
Pain	
Cluster headache, migraine headache, pain, verapamil	2016, 2017, 2019
Trigeminal neuralgia, oxcarbazepine, side effects, epidemiology, etiology	2015, 2015, 2016, 2020, 2021, 2022
Pharmacology	
Baclofen, GABA, spasticity, baclofen withdrawal	2015, 2017, 2018
Pain, amitriptyline	2018, 2020, 2022
Toxic	
Carbon monoxide poisoning, toxicity	2017, 2018, 2020, 2022
Neuro-ophthalmology	
Meniere disease, Vertigo, semicircular canal	2017, 2020, 2022
Dorsal midbrain syndrome, Parinaud syndrome, Gaze paralysis--midbrain tectum	2015, 2017, 2018
Vascular	
CHAD Score, atrial fibrillation. anticoagulation, stroke risk. stroke prevention	2018, 2023, 2023
Gerstmann syndrome, finger agnosia, dominant parietal lobe	2020, 2021, 2022
Thalamus, sensory, stroke, ischemia, Brain stem vascular syndromes	2019, 2019, 2020, 2022
Movement disorders	
Recessive gene, Huntington’s disease	2019, 2020, 2022
Dystonia, basal ganglia, deep brain stimulation, globus pallidus	2017, 2020, 2022
Subthalamic nucleus, hemiballismus, pathophysiology	2015, 2016, 2020, 2022
Autoimmune	
Myasthenia gravis, eye findings, crisis	2018, 2020, 2022, 2023
Lambert-Eaton syndrome, neuromuscular junction, autoimmune disease	2016, 2018, 2020, 2022

Neurosurgery

Neurosurgery is the category with the largest subcategories in the Primary Exam. It is also the category with the most exam questions. Unsurprisingly, the majority of questions in this category are concentrated in cranial neoplastic, cranial vascular, and spinal trauma subcategories. Approximately 14.2% of keywords are tested only once in this category, 12.2% twice, and 73.6% thrice or more during this time period. Table 7 shows the details for high-yield keywords and occurrences. The most common areas tested in this category are pineal region tumors, vestibular schwannomas, carotid disease, degenerative cervical spine disorders, cervical spine trauma, spine deformities, entrapment neuropathies, and pituitary disorders.

**Table 7:** High-yield keywords and occurrences in Neurosurgery.

Neurosurgery keywords	Occurrence years
Cranial - Infection	
Osteomyelitis, infection, skull fracture	2018, 2020, 2022
Infection, cerebral abscess, MR image diagnosis, hereditary disorders	2017, 2018, 2019, 2021
Subdural empyema, infection	2016, 2017, 2019, 2020, 2022
Cranial - Congenital	
Achondroplasia, foramen magnum stenosis, American Academy of Pediatrics Clinical Report	2017, 2019, 2020, 2022
Craniosynostosis, increased intracranial pressure	2017, 2020, 2022
Kleeblattschädel, cloverleaf deformity, synostosis, craniofacial	2018, 2018, 2020, 2022
Craniofacial syndrome, digital fusion anomalies, Apert	2015, 2016, 2017, 2020, 2022
Pfeiffer syndrome, coronal suture synostosis	2019, 2020, 2022
Arachnoid cyst, CT scan and MR imaging characteristics	2017, 2020, 2022
Endoscopic third ventriculostomy, anatomy, complications, hypothalamus	2015, 2019, 2021
Hydrocephalus, myelomeningocele, Chiari II, dysraphism, ventricular shunt malfunction	2016, 2021, 2023
Neurological embryology, benign intracranial masses, lipoma	2017, 2018, 2019
Phakomatoses, subependymal giant cell tumor, tuberous sclerosis, Sturge‐Weber syndrome, NF2	2021, 2020, 2022
Neonatal intraventricular hemorrhage, posthemorrhagic hydrocephalus	2018, 2019, 2020, 2020, 2022, 2022
Cranial - Trauma	
DECRA trial, traumatic brain injury, decompressive craniectomy, surgical technique	2017, 2017, 2019, 2020, 2020, 2022, 2023
Trauma, radiographic assessment, asymptomatic victim, head trauma, Glasgow Coma Scale	2015, 2016, 2023, 2023
Biomarkers, concussion, evaluation, return to sports after mild concussion	2016, 2017, 2018, 2021
Cranial - Tumor	
Craniopharyngioma, diabetes insipidus, pituitary failure, Gamma knife, radiotherapy, BRAF	2017, 2018, 2019, 2020, 2020, 2022, 2022
Pineal region tumor, veins, teratoma, third ventricle, germ cell tumor, suboccipital craniotomy, pediatric brain tumors clinical signs	2015, 2016, 2018, 2019, 2019, 2020, 2020, 2022, 2022, 2022, 2023
Ependymoma, cerebellar mutism, telovelar approach, fourth ventricle surgery	2018, 2020, 2023
Brain metastasis, cerebellar metastasis, breast cancer, leptomeningeal metastases	2016, 2017, 2018
Von Hippel-Lindau disease, hemangioblastoma, endolymphatic sac tumor, autosomal dominant, pheochromocytoma	2016, 2018, 2018, 2020, 2021, 2022
Neurocutaneous syndromes neurofibromatosis type I, tuberous sclerosis complex	2017, 2018, 2019
Primary GBM, secondary GBM, IDH1, p53, PTEN, EGFR, PDGF, Glioma, biomarkers, prognosis	2015, 2015, 2016, 2017, 2019, 2020, 2022
Meningioma, Simpson grade, optic neuropathy, meningioma, foramen magnum, vascular supply, skull base, VEGF, peritumoral edema	2015, 2015, 2017, 2017, 2019, 2020, 2022, 2023, 2023
Vestibular schwannoma, acoustic neuroma, skull base, cranial nerves, facial nerve, surgical approaches, vertical crest, transverse crest	2015, 2017, 2017, 2018, 2018, 2018, 2019, 2019, 2019, 2020, 2021, 2022, 2023, 2023
Paraganglioma, chemodectoma, carotid body tumor, Horner’s syndrome, glomus jugulare tumor	2015, 2016, 2021
Cranial - Vascular	
Decompressive craniectomy, outcome, stroke	2018, 2020, 2021, 2022, 2023
Subarachnoid hemorrhage re-rupture	2017, 2017, 2019, 2020, 2022
Cerebral aneurysm, clipping, anterior clinoidectomy, ophthalmic aneurysm, air-filled cavity	2015, 2016, 2019
Mycotic aneurysm--location	2019, 2020, 2020, 2022, 2022
Arteriovenous malformation, natural history, hemorrhage risk, venous varix	2016, 2017, 2017
Internal carotid artery occlusion, collateral flow, posterior communicating artery	2015, 2016, 2017
Dural arteriovenous fistula, cortical venous reflux	2018, 2018, 2019, 2020, 2020, 2022, 2022, 2023
Venous thrombosis, oral contraceptives, heparin, transverse sinus thrombosis	2015, 2017, 2020, 2021, 2022
Moyamoya disease, cerebral ischemia, genetics, Down syndrome, children, indirect bypass, encephaloduroarteriosynangiosis, pial synangiosis	2017, 2019, 2021
Cranial - Other	
Epidermoid cyst	2017, 2018, 2019
Amnesia, fornix, colloid cyst, choroid plexus, ventricular anatomy	2020, 2022, 2023
Intracranial hypotension, MR-imaging, CSF leak, low‐pressure headache, secondary Chiari	2016, 2020, 2022
Extracranial vascular	
Carotid endarterectomy, stroke, cranial nerve injury, vagus nerve, hypoglossal nerve, high carotid artery bifurcation, intracerebral hemorrhage, angioplasty	2015, 2016, 2017, 2017, 2018, 2019, 2020, 2021, 2021, 2021, 2022, 2023, 2023
Carotid dissection, blunt trauma, vascular, pseudoaneurysm, dissecting aneurysm, internal carotid artery dissection, mitosis, ptosis without anhidrosis	2017, 2018, 2019, 2019, 2021, 2023
Spinal - Degenerative	
Cervical spondylotic myelopathy, laminoplasty, C5 palsy, anterior cervical discectomy, anterior cervical corpectomy, Horner’s syndrome, sympathetic chain, dysphonia, Hoarseness, recurrent laryngeal nerve injury, longus colli retraction	2015, 2015, 2017, 2017, 2017, 2017, 2018, 2019, 2020, 2021, 2021, 2021, 2021, 2022, 2023
C6 radiculopathy, herniated disc C5-6, ruptured cervical disc--signs	2018, 2018, 2019, 2019, 2019
Cervical radiculopathy, peripheral nerve entrapment	2020, 2022, 2023
Spondylolisthesis, lumbar fusion, instability, isthmic spondylolisthesis	2018, 2021, 2021
Spine, lumbar, degenerative spondylolisthesis, guidelines, spinal stenosis	2015, 2016, 2018
Microdiscectomy, minimally invasive surgery, disc herniation	2017, 2018, 2023
Pedicle screw, lamina, superior articular process, transverse process, thoracic spine fixation	2018, 2020, 2022
Spinal - Congenital	
Neurenteric cyst	2015, 2016, 2017, 2018
Spinal - Infection	
Discitis management, osteomyelitis, percutaneous disc space biopsy, spine abscess	2015, 2016, 2016, 2017, 2017, 2018, 2019, 2023
Spinal - Trauma	
Cervical spine trauma, spinal cord injury, classification, halo, ligamentotaxis, DISH, closed cervical traction, jumped facets, spinal cord injury, ASIA, surgical reduction, bilateral jumped facet, cervical teardrop fracture	2017, 2018, 2020, 2021, 2022, 2023, 2023, 2016, 2017, 2020, 2021, 2022, 2023, 2023
NEXUS, cervical fracture, cervical clearance	2020, 2021, 2022, 2023
C1-C2 fusion, type II odontoid fracture, type I odontoid fracture, Hangman’s fracture	2015, 2019, 2020, 2021, 2022, 2023, 2023
Odontoid screw. transverse ligament, elderly, geriatric, osteoporosis, orthosis, Jefferson fracture	2017, 2017, 2017, 2019, 2023
Os odontoideum, return to play	2019, 2020, 2022
Atlantoaxial subluxation, traction reduction, atlantoaxial instability, Down syndrome	2015, 2019, 2021, 2021
Rheumatoid arthritis, atlantoaxial subluxation, spinal deformities	2016, 2017, 2018, 2019, 2021
Hyperextension, central cord syndrome	2016, 2021, 2023
Thoracolumbar injury classification and severity score, anterior approach, flexion-distraction injury	2015, 2016, 2017, 2023
Chance fracture, CT scan image, Lumbar burst fracture	2016, 2020, 2022
Ankylosing spondylitis fracture management, marginal syndesmophytes	2017, 2018, 2018, 2019
Osteoporosis, compression fracture, teriparatide, osteopenia, metabolic bone disease, vertebroplasty, fragility fracture, DEXA (dual energy X‐ray absorptiometry), 25‐hydroxyvitamin D	2015, 2015, 2019, 2020, 2022
Spinal - Tumor	
Spine, tumor, osteoid osteoma	2020, 2020, 2022, 2022
Spinal - Vascular	
Foix-Alajouanine syndrome, spinal cord venous congestion, spinal cord arteriovenous malformation, spinal cord hemorrhage, spinal arteriovenous fistula, type 1 spinal arteriovenous malformation	2015, 2016, 2017, 2017, 2018, 2019, 2019, 2021, 2023
Spinal – Biomechanics	
Bending fatigue, spinal instrumentation, spinal biomechanics, pull out strength, modulus of elasticity strength, load	2016, 2016, 2017, 2019, 2021
Interbody graft, subsidence, bone density, interbody graft alternatives	2015, 2017, 2018
Spinal - Deformity	
Interbody fusion, spino-pelvic parameters, spinal alignment	2015, 2016, 2018, 2019, 2020, 2020, 2021, 2023
HRQOL, adult spinal deformity, lumbar lordosis, pelvic incidence, pelvic incidence (PI), pelvic tilt (PT), sacral slope (SS), lumbar lordosis (LL)	2022, 2022, 2023
Complications - Cranial and Spinal	
Subthalamic nucleus lesion-complications, deep brain stimulation	2015, 2019, 2020, 2021, 2022
Pallidotomy, complications, subthalamic nucleus lesion complications	2015, 2016, 2023
Supplementary motor area syndrome	2015, 2016, 2023
Peripheral nerve	
Entrapment neuropathy	2015, 2016, 2017
Carpal tunnel, median nerve, ulnar nerve, Guyon’s canal, transverse carpal ligament, pancoast tumor, cubital tunnel, pronator teres, transposition, ulnar nerve	2016, 2019, 2019. 2020, 2021, 2022, 2022
Brachial plexus, C5 palsy, paraspinal muscles, posterior cord	2015, 2021, 2023
Interosseus nerve, pronator syndrome, anterior interosseous nerve syndrome, pinch sign	2020, 2021, 2022
Peroneal nerve, foot dorsiflexion. foot eversion, ganglion cyst	2019, 2020, 2020, 2021, 2022, 2022
Pain	
Pain, headache	2015, 2021, 2023
Trigeminal neuralgia, rhizotomy, glycerol, radiosurgery, surgical Rx	2016, 2017, 2018, 2020, 2020, 2020, 2021, 2022, 2022, 2022, 2023
Glossopharyngeal neuralgia, vascular compression	2015, 2015, 2016, 2017, 2019, 2023
Cordotomy, pain, pelvic and visceral pain procedures	2017, 2019, 2021
Dorsal anterior cingulate cortex, cognitive control, affective pain, cingulotomy	2016, 2018, 2019
Intrathecal baclofen, spasticity, Baclofen withdrawal, pump, spasticity	2019, 2020, 2022
Dorsal root entry zone lesion	2017, 2020, 2022
Endocrine	
Apoplexy, pituitary adenoma, trans-sphenoidal, prolactinoma, Hyperprolactinemia	2018, 2019, 2019, 2020, 2021, 2022, 2023
Pituitary surgery complications, SIADH. hyponatremia, stalk effect	2017, 2018, 2018, 2019, 2020, 2022
Cushing syndrome laboratory studies, sphenoid sinus ostium, nonfunctional pituitary tumor, pituitary hormone replacement, adrenal crisis, panhypopituitarism, pituitary tumor, endoscopy, technique, endonasal, optico carotid recess	2016, 2017, 2020, 2022
Surgical technique	
Pituitary stalk, pterional craniotomy	2015, 2016, 2017
Autonomic	
Sympathetic nervous system, hyperhidrosis	2015, 2016, 2020, 2021, 2022
Movement disorders	
Ventral lateral thalamotomy, tremor, Parkinson’s disease	2015, 2017, 2021, 2023
Parkinson disease, globus pallidus internus, deep brain stimulation, microlesion effect	2015, 2016, 2019, 2019, 2020, 2020, 2022, 2022
Essential tremor, deep brain stimulation, ventral intermediate nucleus	2015, 2017, 2018, 2018
Radiosurgery	
Radiation, physics, radiation therapy, radiosurgery, conformity index, glioblastoma	2016, 2018, 2019, 2023
Brain metastasis, stereotactic radiosurgery, craniotomy, whole brain radiation therapy	2017, 2018, 2020, 2021, 2022
Acoustic neuroma, vestibular schwannomas, stereotactic radiosurgery, hearing	2015, 2017, 2018, 2020, 2021, 2022
Endovascular	
AVM embolization, glue, complication avoidance, endovascular technique, venous stasis	2019, 2020, 2021, 2022
Carotid stent, stroke, carotid angioplasty, risk of ischemic stroke, symptomatic patient	2017, 2018, 2019, 2019, 2019
Thrombectomy, stroke, TPA, endovascular, Abciximab	2018, 2021, 2023
Endovascular, ischemic stroke, large vessel occlusion, thrombectomy, DAWN trial	2019, 2020, 2022
Intracranial stenting, intracranial aneurysms, aneurysm, flow diversion	2016, 2018, 2020, 2022
Surgical epilepsy	
Epilepsy, pediatrics, hemimegalencephaly, hemispherectomy	2019, 2020, 2022
Temporal lobectomy, postoperative deficits, mesial temporal lobe epilepsy. hippocampal sclerosis, CN IV-trochlear nerve, mesial temporal lobe	2016, 2019, 2019, 2019, 2021
Temporal lobectomy, optic tract, mesial temporal anatomy, temporal stem, surgical anatomy	2016, 2020, 2022
Epilepsy, surgery, vagal nerve stimulation, seizures	2016, 2020, 2022

Critical care

Critical Care and Core Competencies are the latest entries to the ABNS Primary Exam. Subcategories in Critical Care are fairly intuitive and focused on other systems related to neurosciences and neurosurgery in general. Approximately 12.5% of keywords are tested only once in this category, 15.4% twice, and 72.1% thrice or more during this time period. Table 8 shows the details for high-yield keywords and occurrences. The most common areas tested in this category are surprisingly in wound healing, including nutrition, collagen synthesis, wound hydration, and diabetes.

**Table 8:** High-yield keywords and occurrences in Critical Care.

Critical Care keywords	Occurrence years
Fluid and electrolytes	
Osmolal gap, electrolytes	2016, 2017, 2021
Sodium, pseudohyponatremia, electrolyte problems	2015, 2016, 2017, 2019, 2021
Alkalosis, acidosis-lab measurements, metabolic, respiratory	2015, 2018, 2021
Blood gasses--metabolic acidosis, respiratory	2018, 2020, 2020, 2022, 2022
Hyperkalemia, EKG, hypocalcemia	2015, 2016, 2020, 2022, 2023
Hypomagnesaemia	2016, 2019, 2021
Cerebral salt wasting, hyponatremia, SIADH, vasopressor receptor antagonist	2016, 2017, 2023, 2023
Shock	
Shock, pharmacology	2015, 2017, 2018, 2019, 2023
Shock, critical care, cardiogenic shock	2018, 2022, 2020, 2023
Resuscitation, venous access, transfusion hemodynamic monitoring, fluid responsiveness	2015, 2023, 2023, 2023
Neurogenic shock, spinal shock, baroreceptor reflex	2019, 2020, 2022, 2023, 2023
Blood transfusion reaction, treatment	2015, 2016, 2020, 2022
Hemorrhagic shock	2015, 2020, 2022
Cardiovascular	
Atrial fibrillation, thyrotoxicosis, restarting anticoagulation, CHADS2 score, tachycardia, chronic kidney disease	2016, 2016, 2017, 2017, 2023
Cardiac arrhythmia, atrial flutter, cardiac arrhythmia, beta blocker	2018, 2021, 2023
Echocardiography, myocardial function, cardiac output, hemodynamic monitoring	2015, 2017, 2018, 2019, 2020, 2022, 2023
Arrhythmia, Mobitz I	2016, 2017, 2018, 2023
Cardiac failure, etiology	2015, 2018, 2019, 2023
Hypothermia, outcome, cardiac arrest, indications	2015, 2017, 2019
Ventricular fibrillation, management	2015, 2016, 2017, 2019
Multiple trauma	
Traumatic brain injury, metabolic responses, multiple trauma, hypotension	2016, 2017, 2018, 2020, 2020, 2022, 2022
Post-traumatic epilepsy, status epilepticus, anticonvulsants, seizure prophylaxis	2016, 2017, 2019, 2021
Mannitol, hypertonic saline, traumatic brain injury, hyperosmolar therapy, intracranial hypertension, Pentobarbital, propofol	2017, 2018, 2018, 2019, 2021, 2023, 2023
Brain death, trauma, critical care. brain-stem reflexes, Apnea test	2018, 2021, 2023
Mean arterial blood pressure	2016, 2017, 2018, 2021
Cerebral blood flow, cerebral oxygenation, cerebral perfusion, autoregulation, cerebral blood flow, cerebral metabolism	2018, 2020, 2022
Hyperventilation, intracranial hypertension, neurotrauma	2016, 2017, 2020
Traumatic brain injury, intracranial pressure monitoring, edema, BBB	2017, 2017, 2017, 2018, 2018, 2019, 2021
Meningitis in closed skull fracture, basilar skull fx, CNS infection, guideline	2015, 2019, 2020, 2021, 2022
Complication, dysreflexia, hypertension, spinal cord injury	2019, 2021, 2023
Hematology and coagulopathies	
Dabigatran, anticoagulation reversal, direct thrombin inhibitor	2017, 2018, 2019
Coagulopathies, closed head injury	2016, 2016, 2017, 2018, 2019
Hematology and coagulopathies	2018, 2020, 2022
Praxbind, idarucizumab, PTT, abciximab, platelets, phosphodiesterase inhibitors	2017, 2018, 2023
Anticoagulation, novel oral anticoagulants, intracerebral hemorrhage, reversal	2017, 2020, 2021, 2022, 2023
Heparin, mechanism, enoxaparin, anti-coagulation, protamine sulfate	2015, 2016, 2017, 2020, 2021, 2022
Coagulation, hemostasis, coagulation factors: PTT	2015, 2017, 2019, 2020, 2022
DDAVP, hemorrhage, platelets, warfarin, PCC, vitamin K, idarucizumab, clopidogrel	2017, 2020, 2020, 2021, 2022, 2022
Deep venous thrombosis, anticoagulation	2015, 2021, 2023
Clopidogrel, mechanism of action	2015, 2016, 2019, 2020, 2022, 2023
Nutrition	
Nutrition, traumatic brain injury, basal energy expenditure post-trauma	2017, 2018, 2020, 2022, 2023
Pulmonary	
Acute respiratory distress syndrome, critical care, ECMO, pulmonary edema	2018, 2018, 2019, 2019, 2020, 2021, 2022, 2023, 2023
Pulmonary, ventilator acquired pneumonia	2015, 2016, 2017, 2020, 2022, 2023
Respiratory failure-aspiration pneumonia, etiology--nosocomial pneumonia	2015, 2017, 2021
Mechanical ventilation, pneumothorax, tube thoracostomy, Dobhoff	2015, 2017, 2019, 2021, 2020, 2022, 2023
Pulmonary ventilation---adequacy, PEEP, excessive, FiO_2_, pulmonary atelectasis, oxygenation	2015, 2016, 2020, 2021, 2022
Critical care, endobronchial intubation, pulmonary	2015, 2016, 2017, 2020, 2022
intubation and hyperventilation, intracranial pressure	2016, 2021, 2022
Motor neuropathy, Guillain-Barre syndrome, vital capacity	2016, 2020, 2022
Respiratory failure, spinal cord injury	2020, 2022, 2023
Venous air embolism detection	2020, 2022, 2023
Aerosol, disease transmission, aerosolization, oxygen therapy	2020, 2022, 2023
Cheyne-Stokes respirations: apnea, hyperventilation	2019, 2020, 2022
Endocrine	
Steroids in neurological critical care	2015, 2021, 2023
Thyroid disease--treatment, hyperthyroidism, ECG, thyroid function tests	2017, 2020, 2022
Multiple endocrine neoplasia syndrome	2017, 2018, 2020, 2022
Aldosteronism--signs, Cushing disease, endocrine, pituitary, diagnostic testing	2015, 2021, 2023, 2023
Hyponatremia, pituitary surgery, SIADH	2020, 2020, 2021, 2022, 2022
Adrenal complication, etomidate, side effect	2016, 2019, 2019,2021
Hyperparathyroidism - diagnosis	2016, 2019, 2021, 2023
Diabetes insipidus, DDAVP, parasellar tumor	2019, 2020, 2022
Gastrointestinal	
Acute upper gastrointestinal bleeding, acute gastritis	2017, 2019, 2021
Genito-urinary	
Acute renal failure, acute tubular necrosis, oliguria	2018, 2019, 2021
Pharmacologic	
Anticonvulsant, pharmacology, hepatic failure	2017, 2020, 2022
Status epilepticus, benzodiazepines, lorazepam, diazepam	2018, 2020, 2022, 2023
Neuroleptic malignant syndrome, D2 receptor blockade, butyrophenones	2017, 2017, 2018, 2018, 2020, 2021, 2022
Propofol, hyperlipidemia, pharmacology, propofol infusion syndrome, rhabdomyolysis	2016, 2018, 2019, 2020, 2021, 2022, 2023
Remifentanil	2015, 2018, 2020, 2022
Hyperkalemia, spinal cord injury, succinylcholine	2018, 2020, 2022
Infection and immunologic	
Intravenous fluids, resuscitation, sepsis, shock	2015, 2016, 2016, 2017, 2019, 2021
Fever, sepsis, steroids, treatment	2018, 2020, 2022, 2023
Parenteral alimentation, sepsis	2019, 2021, 2023
Tuberculosis, pyridoxine, isoniazid therapy	2015, 2016, 2017, 2023
Toxicology	
Epilepsy, hyperammonemia, drug toxicity	2017, 2019, 2021, 2023
Subacute cerebellar degeneration, alcoholism	2016, 2018, 2019, 2021, 2023
Wound healing	
Nutrition, collagen synthesis, wound hydration, diabetes	2015, 2016, 2016, 2016, 2017, 2018, 2019, 2020, 2020, 2022, 2022, 2023
Anesthesia	
Malignant hyperthermia, inhalation, intracellular calcium, perioperative	2015, 2017, 2019, 2023
Opioid metabolism, pharmacology	2019, 2020, 2022
Neuroanesthesia, pharmacology, intravenous anesthesia, subarachnoid hemorrhage	2019, 2020, 2022
Cerebral metabolism	
SAH, vasospasm, delayed ischemic neurological deficit, vasopressor	2015, 2016, 2016, 2018, 2021
Atrial fibrillation, hypervolemia, fluid balance, subarachnoid hemorrhage	2019, 2020, 2022
Subarachnoid hemorrhage, cardiac function, cardiac injury, Terson	2017, 2020, 2020, 2021, 2022, 2022
Acute stroke, tissue plasminogen activator, tPA contraindications	2017, 2018, 2020, 2022
Venous sinus thrombosis, increased risk	2015, 2018, 2019, 2021
Aneurysm, airway management, hydrocephalus	2017, 2019, 2023
Other	
Venous air embolism detection	2018, 2021, 2023
Stellate ganglion block, locked-in syndrome	2016, 2021, 2023

Core competencies

The Core Competencies category is the smallest section in the Primary Exam, only slotted for seven questions out of 750 each year. It follows Accreditation Council for Graduate Medical Education’s (ACGME) Milestones and ABNS added patient safety and ethics. Most of the questions can be found under the subcategory of Practice-Based Learning and Improvement.

## Discussion

According to the ABNS, the distributed annual Primary Exam Keywords are to help program directors determine the “strength and weakness of their program.” Our residents usually start taking the Primary Exam as PGY2s, and most of them pass it on their first try. Because most of our residents take the Primary Exam for credit and pass them as PGY4s, they usually take the Primary Exam three times during residency; rare, occasional, and exceptional residents have passed them on their second try. ABNS Primary Exam has a relatively high passing rate (>90%) among first-time credit test takers when compared to other subspecialties [3-5]. Not much has been published about the ABNS Primary Exam except for its relationship with the United States Medical Licensing Exam (USMLE) [6,7]. In view of our background, we created a database using the keywords from 2015 to 2023 as a template for our didactics. The database has evolved into our informal knowledge-based curriculum. According to ACGME program requirements for neurosurgery updated in July 2022, the curriculum “must contain a set of program aims consistent with the Sponsoring Institution’s mission, the needs of the community it serves, and the desired distinctive capabilities of its graduates.” Under medical knowledge, “residents must demonstrate competence in their knowledge of neurosurgical emergencies; treating neurosurgical conditions: cerebrovascular disorders, functional neurosurgery, neurocritical care, neuro-oncology, pain, pediatric neurological surgery, peripheral nerve disorders, spinal disorders and trauma. Different medical practice models and delivery systems and how to best utilize them to care for an individual patient; and, study design and statistical methods. All residents tracking towards ABNS certification must pass the ABNS primary examination before completing the program” [8].

The lists of keywords are quite extensive and comprehensive including both basic and clinical neurosciences. Even though we listed frequently tested and what we consider high-yield keywords, there are a significant number of keywords tested once or twice during 2005-2023 that should be considered as well. It should also be noted that each set of keywords contains percentages of correct among credit candidates and self-assessment test takers which we did not take into account in our study. The ABNS does not instruct individual programs on how to approach or use these keywords. We have expanded our keywords database by inserting comments on the spreadsheet itself from searches using Google Search or ChatGPT. The more precise or specific the keywords, the better the result in terms of information. For instances where images are needed, we linked the keywords to specific URLs. Our residents are encouraged to self-study and add content to the database. There are no specific trends in looking at the keywords and occurrences from 2015 to 2023. It is not uncommon to see a keyword tested for three consecutive years, take a hiatus, and then resurface a few years later. It is also very common to see a certain keyword tested several times in the same year in the same category or in other categories. There are also certain keywords tested every year. Most of the highest yielded keywords are fairly practical in the day-to-day practice of neurosurgery. Most of the keywords tested are often repeated more than three times during this period of time (58.1-82.2%), with the lowest repeat rate in Neurosciences and Neurology and the highest in Neuroradiology.

There are many ways to approach the Primary Exam as it is quite individualized. Most residents use commercially available review books and sample tests while others may take courses sponsored by the Congress of Neurological Surgeons such as Self-assessment in Neurological Surgery (SANS) which has evolved over the years [9-11]. Compared to neurosurgery, general surgery has many more options in terms of online resources. One example is the concept of Question Bank which has shown mixed results over the years [12,13]. Not many resources and references are found to be dedicated to the board exam keywords and such may be due to their inherent limitations. For example, there is no way to predict the exact exam questions and answers based on published keywords. Keywords can also be misleading and interpreted differently depending on the individual. Grouping of the keywords may also be subjective, and we analyzed the most recent ABNS Primary Exams keywords, i.e., 2015-2023, and not their entirety. Keywords for next year’s exam may be very different from what we presented. Because the overall pass rate for the Primary Exams is high at over 90%, keywords themselves may add little or no value to most test takers.

## Conclusions

We have organized, grouped, and tabulated keywords distributed by the ABNS for Primary Exams administered from 2015 to 2023. Throughout the years, many keywords are repeated which may reflect the emphasis of ABNS on certain areas in neurosurgery for residents to be focused on. Neurosurgery residency program directors and residents can consider the compiled list as a reference and guide.
